# Characterising the urinary acylcarnitine and amino acid profiles of HIV/TB co-infection, using LC–MS metabolomics

**DOI:** 10.1007/s11306-024-02161-8

**Published:** 2024-08-03

**Authors:** Charles Pretorius, Laneke Luies

**Affiliations:** Human Metabolomics, North-West University, Potchefstroom Campus, Private Bag X6001, Box 269, Potchefstroom, 2520 South Africa

**Keywords:** HIV/TB co-infection, Metabolomics, Acylcarnitines, Amino acids, 5-HIAA, LC–MS

## Abstract

**Introduction:**

The human immunodeficiency virus (HIV) and tuberculosis (TB) co-infection presents significant challenges due to the complex interplay between these diseases, leading to exacerbated metabolic disturbances. Understanding these metabolic profiles is crucial for improving diagnostic and therapeutic approaches.

**Objective:**

This study aimed to characterise the urinary acylcarnitine and amino acid profiles, including 5-hydroxyindoleacetic acid (5-HIAA), in patients co-infected with HIV and TB using targeted liquid chromatography mass spectrometry (LC–MS) metabolomics.

**Methods:**

Urine samples, categorised into HIV, TB, HIV/TB co-infected, and healthy controls, were analysed using HPLC–MS/MS. Statistical analyses included one-way ANOVA and a Kruskal-Wallis test to determine significant differences in the acylcarnitine and amino acid profiles between groups.

**Results:**

The study revealed significant metabolic alterations, especially in TB and co-infected groups. Elevated levels of medium-chain acylcarnitines indicated increased fatty acid oxidation, commonly associated with cachexia in TB. Altered amino acid profiles suggested disruptions in protein and glucose metabolism, indicating a shift towards diabetes-like metabolic states. Notably, TB was identified as a primary driver of these changes, affecting protein turnover, and impacting energy metabolism in co-infected patients.

**Conclusion:**

The metabolic profiling of HIV/TB co-infection highlights the profound impact of TB on metabolic pathways, which may exacerbate the clinical complexities of co-infection. Understanding these metabolic disruptions can guide the development of targeted treatments and improve management strategies, ultimately enhancing the clinical outcomes for these patients. Further research is required to validate these findings and explore their implications in larger, diverse populations.

**Supplementary Information:**

The online version contains supplementary material available at 10.1007/s11306-024-02161-8.

## Introduction

In the landscape of global infectious diseases, tuberculosis (TB) and human immunodeficiency virus (HIV) present formidable challenges, having long been recognised as leading causes of morbidity and mortality worldwide. Even with significant advances in medical science and public health, these diseases continue to exert a substantial burden. Prior to the disruption caused by the COVID-19 pandemic, TB was the top cause of death from a single infectious agent, responsible for approximately 1.3 million deaths in 2023, excluding an additional 167,000 deaths among HIV-positive individuals. Meanwhile, HIV remains a critical public health issue, with about 1.5 million new infections occurring in 2023, underscoring its ongoing transmission dynamics (Joint United Nations programme on HIV/AIDS, 2023; Olivier & Luies, 2023; World Health Organization, 2023).

The co-infection of HIV and TB exacerbates the complexity of both diagnoses and treatment. Patients infected with HIV are about 20 times more likely to contract *Mycobacterium tuberculosis* (*Mtb*), the pathogen responsible for TB, compared to those without HIV. Conversely, TB can accelerate the progression of HIV by stimulating an immune response that favours HIV replication and exacerbates the patient’s overall health deterioration (Liebenberg et al., 2021; Saharia & Koup, 2013).

This syndemic—a synergistic epidemic involving two or more afflictions that exacerbate the burden or severity of each—necessitates a nuanced understanding of the metabolic interplays at work. Metabolomics, which involves the comprehensive study of small molecule metabolites within an organism, offers a promising approach to elucidate these interactions. Through either targeted or untargeted analyses, metabolomics provides insights into metabolic disruptions caused by diseases, potentially identifying novel differentiating metabolites for early diagnosis and efficient management (Liebenberg et al., 2021).

In HIV/TB co-infection, significant metabolic pathways include those involving acylcarnitines and amino acids (Herbert et al., 2023), which are crucial for energy metabolism and immune function, respectively. Acylcarnitines—derived from the conjugation of carnitine with fatty acids—are essential in the transport of fatty acids into mitochondria for β-oxidation, a critical energy-releasing process. Alterations in acylcarnitine profiles have been observed in TB and may be further complicated by HIV co-infection (Melone et al., 2018; Qu et al., 2016; Schooneman et al., 2013). Furthermore, amino acids play essential roles in protein synthesis and serve as precursors for metabolites which have substantial effects on neurobiological processes and immune regulation (Roth et al., 2021). Tryptophan metabolism, affecting metabolites like serotonin and kynurenine, is notably affected in patients with HIV and TB (Luies & Loots, 2016; Munn & Mellor, 2013; Sitole et al., 2019; Weiner et al., 2012). To this end, the breakdown product of serotonin, 5-hydroxyindoleacetic acid (5-HIAA), is a promising differentiating metabolite in various disease states, reflecting the activity of the serotonin metabolic pathway (Clark et al., 2017). Indeed, decreased serotonin levels have been reported in HIV (Peltenburg et al., 2018) and TB (Cho et al., 2020) patients.

Given the critical need to better understand these metabolic processes in co-infected patients, this study aims to characterise the urinary acylcarnitine and amino acid profiles, including 5-HIAA, of patients with HIV/TB co-infection using targeted liquid chromatography mass spectrometry (LC–MS) metabolomics. By delineating these metabolic profiles, the research seeks to uncover potential pathways that could be targeted for therapeutic intervention, thereby improving treatment strategies and clinical outcomes for this vulnerable patient group.

## Materials and methods

### Sample collection and demographics

Urine samples were collected between 2015 and 2018 under established protocols (Bi et al., 2020) by the Desmond Tutu HIV Foundation and the South African Tuberculosis Vaccine Initiative (SATVI) at the University of Cape Town (UCT). Patients were not necessarily fasting, as sampling was done when individuals visited the clinic upon first diagnosis or a returning clinic visit, at which point they were recruited for the study. These anonymised samples were then transported to the North-West University’s (NWU) Focus Area Human Metabolomics and stored at − 80 °C. Both male and female participants, ranging in age from 18 to 69 and residing in the Masiphumelele and Ocean View Townships of Cape Town, South Africa, were meticulously chosen to minimise variability in *Mtb* strains and to avoid confounding variables. While variability in *Mtb* strains was not specifically tested, confining our study population to a defined geographical area helps to minimise strain variability. This approach was crucial in ensuring a homogeneous study population, thereby enhancing the robustness of our findings related to HIV/TB co-infection. To avoid confounding variables, participants completed questionnaires that collected information on other illnesses, medication intake, and pregnancy/lactation. Participants were organised into four groups based on their confirmed serum HIV and TB status—HIV-only (*n* = 7), TB-only (*n* = 41), HIV/TB co-infected (*n* = 9), and healthy controls (*n* = 32). TB was diagnosed through GeneXpert and HIV via dual rapid antibody tests, in accordance with South African and World Health Organization (WHO) standards. There was no subsequent anonymous re-testing for HIV on the collected samples. All subjects were treatment naïve, and individuals diagnosed with HIV and/or TB were directed to relevant medical services. Details on the cohort demographics, including T cell counts and viral loads for those HIV-positive, are summarised in Table 1. It is important to note that the samples were provided by local township clinics specifically for research purposes, with proper ethical approval and patient consent. According to WHO guidelines, these clinics do not routinely perform CD4 T cell count and viral load testing when diagnosing HIV. Instead, the WHO guidelines recommend two consecutive positive HIV tests for diagnosis rather than regular CD4 and viral load assessments.

**Table 1:** Cohort demographics and clinical information

	HC	TB-only	HIV-only	HIV/TB
No. of patients (%)	32 (36.0)	41 (46.1)	7 (7.9)	9 (10.1)
Age (years), mean ± (range)	37 ± 11.2	35 ± 12.2	37 ± 4.7	33 ± 5.8
Sex, female:male ratio	20:12	10:31	3:4	3:6
Smokers in the group (%)	12 (37.5)	25 (61.0)	3 (42.9)	3 (33.3)
CD4 cell count (cells/mm^3^ blood), mean ± SD	N/A	N/A	269^#^ ± 119.4	135.4 ± 140.2
Viral load (copies/mL), range*	N/A	N/A	Unavailable	< 20–124

HC, healthy controls; HIV, human immunodeficiency virus, TB, tuberculosis; SD, standard deviation, CD4, cluster of differentiation 4; N/A, not applicable

*The viral load was not available for all patients; data shown here do not represent the entire group

^#^This average is based on only two samples for which this data was available

Given the constrained sample sizes, particularly for the HIV-positive (*n* = 7) and HIV/TB co-infected (*n* = 9) groups, influenced by the WHO’s “test-and-treat” policy, the exploratory nature of this study is geared more towards providing a foundational understanding and shaping future inquiries rather than establishing definitive new biomarkers.

### Reagents and chemicals

Isotopically labelled internal standards for the acylcarnitine method were purchased from LGC Standards (Midrand, South Africa). Standards included deuterium-labelled L-carnitine-d3 hydrochloric acid (HCl; C0_ISCas: 350818-62-1), acetyl-L-carnitine-d3 HCl (Cas: 1334532-17-0), propionyl-L-carnitine-d3 HCl (Cas: 1334532-19-2), butyryl-L-carnitine-d3 HCl (Cas: 1334532-21-6), isovaleryl-L-carnitine-d9 HCl (Cas: 1334532-23-8), hexanoyl-L-carnitine-d3 HCl (Cas: 2483831-95-2), octanoyl-L-carnitine-d3 HCl (Cas: 1334532-24-9), decanoyl-L-carnitine-d3 HCl (Cas: 2483831-87-2), dodecanoyl-L-carnitine-d3 HCl (Cas: 2687960-76-3), myristoyl-L-carnitine-d9 HCl (Cas: 1334532-25-0), palmitoyl-L-carnitine-d3 HCl (Cas: 1334532-26-1), and octadecanoyl-L-carnitine-d3 HCl (Cas: 2245711-27-5). ^13^C-labelled 5-hydroxyindole-3-acetic acid-^13^C3 (5-HIAA_IS; Cas: 51-16-0) was purchased from Toronto Research Chemicals (Toronto, Canada) and used as the internal standard for the 5-HIAA method. Unlabelled standards used during method validation were purchased from Merck (Darmstadt, Germany), and for the acylcarnitine method, included acetyl-L-carnitine (C2; Cas: 3040-38-8), propionyl-L-carnitine (C3; Cas: 20064-19-1), butyryl-L-carnitine (C4; Cas: 25576-40-3), isovaleryl-L-carnitine (C5; Cas: 31023-24-2), hexanoyl-L-carnitine (C6; Cas: 22671-29-0), octanoyl-L-carnitine (C8; Cas: 25243-95-2), decanoyl-L-carnitine (C10; Cas: 1492-27-9), dodecanoyl-L-carnitine (C12; Cas: 2466-77-5), and 5-HIAA (Cas: 54-16-0) for the 5-HIAA method. Synthetic urine from Industrial Analytical (Pty) Ltd (a subsidiary of LGC Standards; Midrand, South Africa) was used during the validation of the acylcarnitine method. LC–MS grade solvents, namely acetonitrile (ACN; Cas: 75-05-8), methanol (MeOH; Cas: 67-56-1), and water (H_2_0; Cas: 7732-18-5), were from Honeywell, Burdick & Jackson, supplied by Anatech (Randburg, South Africa). Formic acid (Cas: 64-18-6) used as the mobile phase modifier, as well as acetyl-chloride (Cas: 75-36-5) and 1-butanol (Cas: 71-36-3) used for butylation were also from Merck. All reagents used for the amino acid analysis, including the mobile phases, internal standards, calibrators, and quality controls, were included in the ChromSystems MassChrom® Amino Acid Analysis kit (supplied by Separations, Johannesburg, South Africa).

### Urine sample preparation

#### Sample preparation for acylcarnitine analysis

Quality control (QC) and patient urine samples were prepared by adding 100 μL of an acylcarnitine isotope working solution (Table S1) and 30 μL of ACN to 10 μL of each sample in a microcentrifuge tube. After centrifugation at 12,000×*g* for 10 min at 4 °C, the supernatants were transferred to vials, dried under nitrogen, then butylated using 200 μL of 3 N butanolic HCl for 30 min at 60 °C and vortexed for 20 s. The butylated samples were dried again under nitrogen and reconstituted in 100 μL of ACN:H_2_O (1:1) for 30 min, and vortexed for another 20 s before being aspirated into a pulled vial insert for high-performance liquid chromatography tandem mass spectrometry (HPLC–MS/MS) analysis (Smith & Matern, 2010).

#### Sample preparation for 5-HIAA analysis

QC and patient samples were prepared as follows: Each urine sample (50 μL) was supplemented with 50 μL of a 51.1 μmol/L 5-HIAA-^13^C3 isotopically labelled internal standard working solution in ACN:H_2_O (1:1). Samples were centrifuged at 12,000×*g* for 10 min at 4 °C, and the supernatant was transferred to a pulled insert in a vial for subsequent analysis (Clark et al., 2017).

### HPLC–MS/MS analyses

#### Acylcarnitines and 5-HIAA analyses

All experimental samples were randomised and divided into six batches, with a pooled QC aliquot assigned to each batch. Each batch was prepared separately and analysed twice using two validated methods specifically adapted for this study; one for acylcarnitine analysis (Smith & Matern, 2010), and the other for 5-HIAA analysis (Clark et al., 2017). Before analysing each batch, a blank sample containing H_2_O was injected to equilibrate the instrument. This blank sample was re-injected and analysed midway and post-batch to assess potential carry-over effects. QC samples were analysed at the beginning, middle, and end of each batch to gauge between-batch reproducibility, with the relative standard deviation (RSD%) measured for each analyte. The run time for acylcarnitine analysis in dynamic multiple reaction monitoring (dMRM) mode was 32 min, while the 5-HIAA analysis, set to MRM mode, took 13 min per sample. The duration of the analyses was six days for acylcarnitines (one batch daily) and three days for 5-HIAA (two batches daily). The analytical equipment included an Agilent 1260 Infinity II autosampler, 1290 Infinity binary pump, and a 6470 MS/MS with positive electrospray ionisation, with an injection volume of 1 μL for both methods. Details regarding the HPLC–MS/MS chromatography (Table S2), mass spectrometer source conditions (Table S3), and MRM/dMRM transitions (Table S4) are provided in the supplementary material.

Additionally, a principal component analysis (PCA) scores plot created by MetaboAnalyst (version 5.0) was employed to visually inspect the clustering of QC samples compared to patient samples, specifically for the acylcarnitine analysis (Fig. S1). This visualisation aids in confirming the analytical consistency and distinct metabolic profiles between the groups.

#### Amino acid analysis

Amino acids were analysed using the ChromSystems MassChrom® Amino Acid Analysis kit following the manufacturer’s instructions, which facilitates the quantification of 52 amino acids and creatinine in urine. Sample preparation was automated on a MassSTAR robot, which added 50 μL of Internal Standard Mix (order no. 75246) and 600 μL of Dilution Buffer (order no. 75205) to 20 μL of urine/QC sample in a 96-well plate. After centrifugation at 2000×*g* for 5 min at room temperature, 200 μL of supernatant was transferred to a sealed Collection Plate (order no. 75058) for analysis. Each sample had a run time of 19.1 min. Analysis was conducted in three separate batches, with QC samples analysed in triplicate at the start, middle, and end of each batch to assess between-batch reproducibility, with RSD% calculated for each analyte. The HPLC–MS/MS system employed an Agilent Infinity 1290 binary pump paired with a 6470 MS/MS, using positive electrospray ionisation in MRM mode. The analysis utilised the kit’s proprietary analytical column (order no. 75100).

### Data processing

Data acquisition and quantification were performed using Agilent MassHunter Data Acquisition (version 10.0) and MassHunter Quantitative for QQQ software (version 10.0), respectively. For acylcarnitines and 5-HIAA, absolute quantification was achieved using calibration curves constructed from reference standards and their isotopically labelled analogues (Table S5). Metabolite concentrations below the limit of detection were recorded as zero values, and results were normalised to creatinine concentrations (determined using the ChromSystems MassChrom® Amino Acid Analysis kit), expressed as μmol/mmol/creatinine.

### Statistical analysis

MetaboAnalyst (version 5.0) facilitated the multivariate and univariate statistical evaluation of the acylcarnitine and amino acid analyses. All zero values in the datasets were replaced by one-fifth of the lowest recorded value for each respective feature to handle non-detections. Both the acylcarnitine and amino acid datasets underwent log-transformation and auto-scaling to normalise data distribution. A kernel density plot confirmed a normal distribution, justifying the use of parametric tests for these datasets. Multivariate analyses included PCA to reduce the dimensions of the complex data, effectively summarising the dataset into simpler, interpretable components. This approach is valuable for uncovering patterns, detecting trends, or identifying outliers within the dataset (Eriksson et al., 2013). For univariate analysis, one-way analysis of variance (ANOVA) was utilised to explore significant variations in acylcarnitines and amino acids among the comparative groups. The data were corrected for multiple testing, and variables were considered significant if they had both a false discovery rate (FDR) and *p*-value ≤ 0.05. Fisher’s least significant difference was used for post-hoc analysis.

Regarding the 5-HIAA data, the zero values were also replaced by one-fifth of the lowest 5‑HIAA-concentration. Statistical analysis for the 5-HIAA data was performed using IBM SPSS Statistics [version 29.0.2.0 (20)]. No transformation or scaling was performed on this dataset due to its simplicity. Descriptive statistics, including histograms, skewness, and kurtosis, indicated that the data was skewed. Consequently, a non-parametric Kruskal–Wallis test was used for the 5-HIAA data analysis, with a *p*-value ≤ 0.05 considered significant.

## Results

### Assessment of data quality

The quality of patient sample data was assessed using pooled QC urine samples analysed before, during, and after each analytical batch to mitigate significant instrumentation or analytical errors. PCA of the acylcarnitines (Fig. S1) showed no trends indicating analyte variation related to run-order, suggesting the absence of within-batch drift. The RSD% values for between-batch QC samples remained below the recommended 15% cutoff for targeted analyses, indicating minimal batch effects and reliable data quality (Food & Drug Administration, 2018). All acylcarnitine and 5-HIAA concentrations were predominantly above their detection and quantification limits (Table S6). In terms of the amino acids analysed, only 37 were detected in 50% or more of all samples and thus were included in the statistical analysis.

### Multivariate analysis

PCA scores plots for acylcarnitine and amino acid analyses, when excluding QC samples, showed no distinct separation between the cohorts (Fig. S2), indicating limited utility for further differentiating metabolites selection. This lack of significant distinctions between groups could be attributed to the relatively small size of the cohorts, which often necessitates a larger sample size for more reliable results. Considering these constraints, the findings from the multivariate analysis were not utilised to identify significant differentiating metabolites.

### Univariate analysis

#### Acylcarnitines

While the ANOVA did not find any acylcarnitines significantly altered by HIV, TB, or HIV/TB co-infection, there is a noticeable trend of increased medium-chain acylcarnitine levels in the TB and HIV/TB co-infected groups. Mean concentrations for all medium-chain acylcarnitines [hexanoyl-L-carnitine (C6), octanoyl-L-carnitine (C8), decanoyl-L-carnitine (C10) and dodecanoyl-L-carnitine (C12)] in these cohorts were higher than in the uninfected control group. The ANOVA p-values for medium-chain acylcarnitines were notably lower (see Table 2), especially for C6 (*p* < 0.05). However, their FDR values exceeded the acceptable threshold of 0.05, possibly due to the small sample sizes in some groups and the closely distributed acylcarnitine levels between cohorts, which may have influenced statistical power (Tong & Zhao, 2008).

**Table 2:**
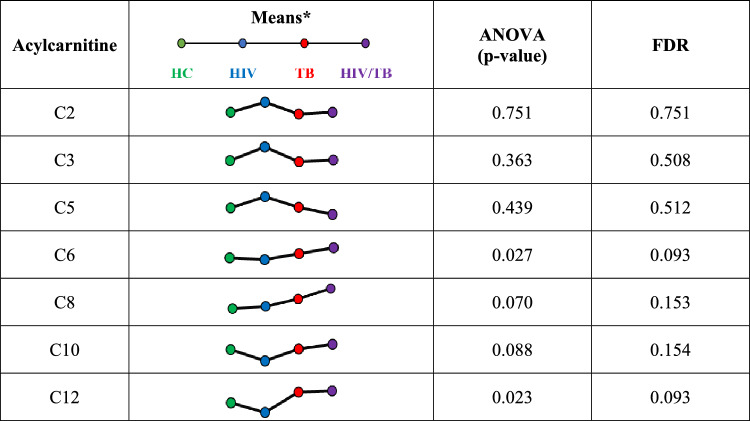
Mean log-transformed acylcarnitine concentrations and statistical analysis

ANOVA, analysis of variance; FDR, false discovery rate; C2, acetyl-L-carnitine; C3, propionyl-L-carnitine; C5, isovaleryl-L-carnitine; C6, hexanoyl-L-carnitine; C8, octanoyl-L-carnitine; C10, decanoyl-L-carnitine; C12, dodecanoyl-L-carnitine

*These trendlines do not represent paired data relationships but rather serve as a general trend visualisation tool

#### Amino acids

The statistical analysis of amino acids included ratios such as glutamine/glutamic acid, kynurenine/tryptophan, phenylalanine/tyrosine, and saccharopine/lysine. ANOVA revealed significant differences in 16 individual amino acids and several ratios across all experimental groups. Generally, amino acid levels were decreased in the HIV-only group and elevated in the TB-only group relative to healthy controls (Table S7).

**Fig. 1:**
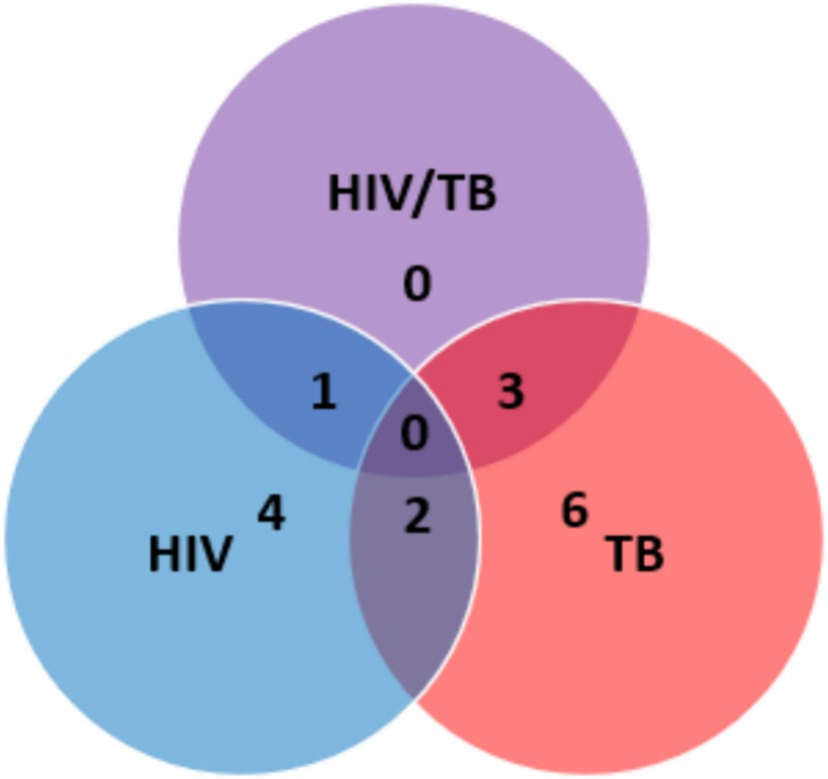
Venn diagram of altered amino acids across the cohorts. This Venn diagram illustrates the unique and shared amino acid alterations in HIV, TB, and HIV/TB co-infected cohorts, highlighting the distinct metabolic impacts of these disease states. *HIV* human immunodeficiency virus only group, *TB* tuberculosis only group, *HIV/TB* co-infected group

Figure 1 presents a Venn diagram that illustrates the unique and shared amino acid alterations across the HIV, TB, and HIV/TB co-infected states, highlighting the distinct metabolic impacts of these conditions. In the HIV group, significant decreases were noted in argininosuccinic acid, phosphoethanolamine, ethanolamine, glutamine, and the glutamine/glutamic acid ratio. The TB cohort exhibited the most pronounced disruptions in amino acid metabolism, with increases in the kynurenine/tryptophan ratio, kynurenine, leucine, arginine, carnosine, and asparagine, alongside a decrease in 3-methylhistidine.

No unique alterations were observed in the HIV/TB co-infected group, although both the HIV and TB groups exhibited significant changes in isoleucine and α-aminoadipic acid levels. The co-infected and HIV groups showed reductions in tryptophan levels, whereas increases in the saccharopine/lysine ratio, hydroxykynurenine, saccharopine, and a decrease in glycine were noted in the HIV/TB co-infected and TB groups. Table 3 summarises these findings, listing the 19 significantly altered amino acids and amino acid ratios along with their statistical analysis results. Notably, only 16 out of the 37 amino acids included in the statistical analysis, and three of the ratios, were significantly altered.

**Table 3:**
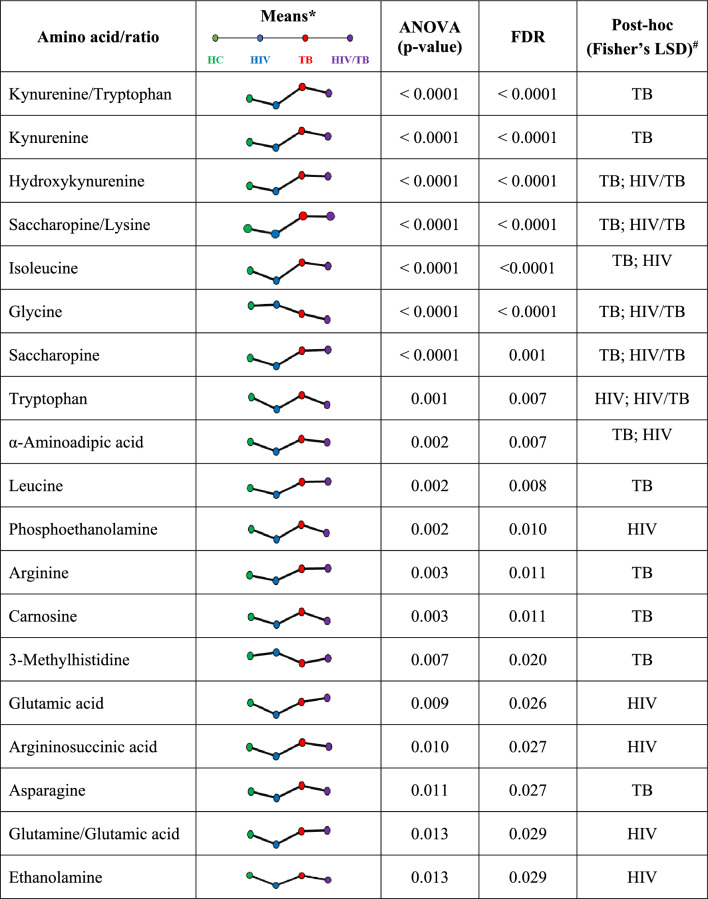
Statistical analysis results of significantly altered amino acids, arranged according to *p*‑value

HC, healthy controls; HIV, HIV-positive; TB, TB-positive; HIV/TB, HIV/TB coinfected; ANOVA, analysis of variance; FDR, false discovery rate; Fisher’s LSD, Fisher’s least significant difference

*These trendlines do not represent paired data relationships but rather serve as a general trend visualisation tool

^#^Diseased states in which amino acids were considered significantly altered when compared to the healthy control group by Fisher’s LSD post-hoc analysis

#### 5-HIAA

**Fig. 2:**
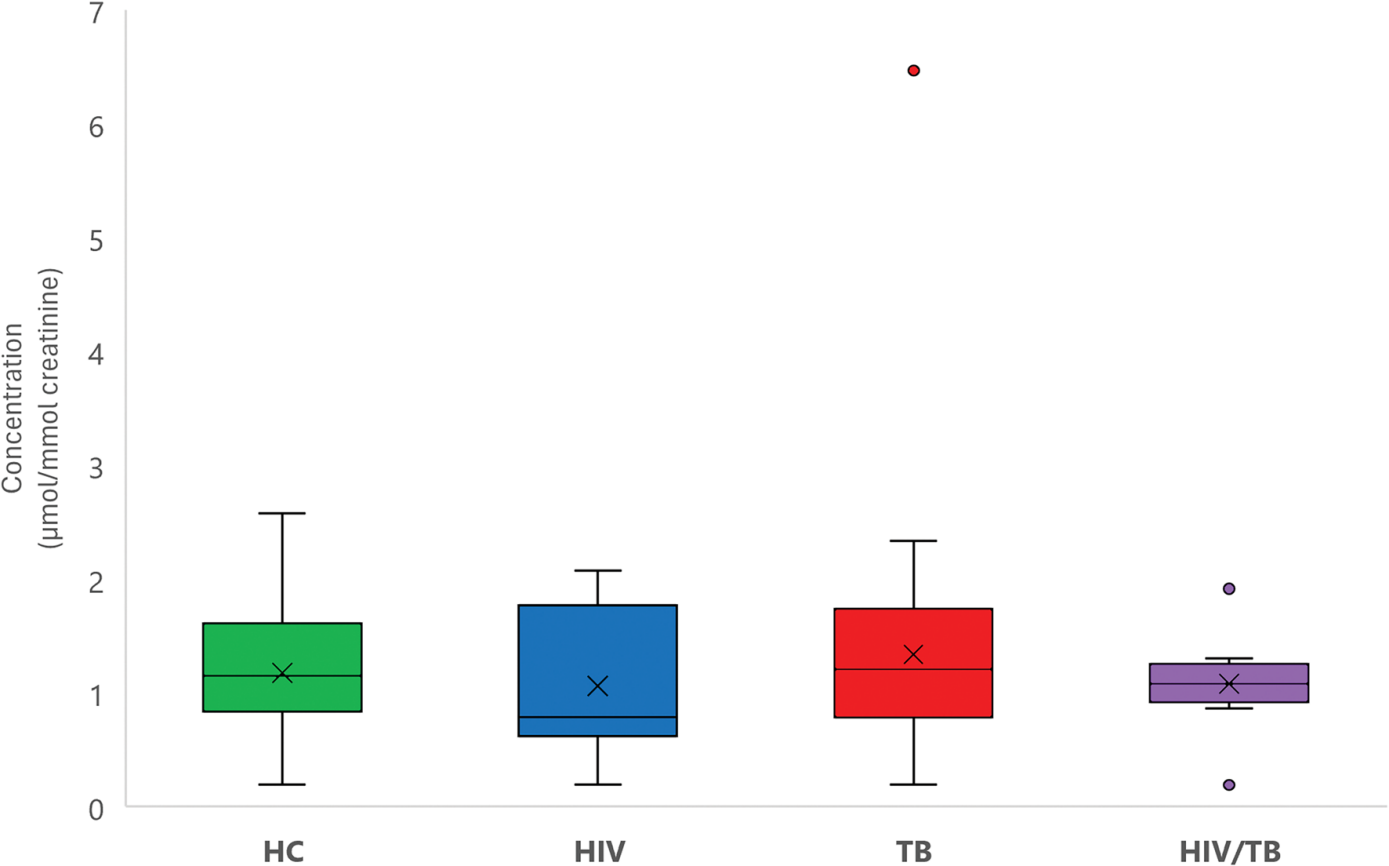
Box and whiskers plot of urinary 5-hydroxyindoleacetic acid (5-HIAA) levels. This plot compares urinary 5-HIAA levels among healthy controls (HC; green), as well as HIV (blue), TB (red), and HIV/TB co-infected (purple) groups, with means indicated by a cross. It demonstrates that there are no significant differences in 5-HIAA levels across the groups

The Kruskal–Wallis test (*p* = 0.791) showed no significant differences in urinary 5-HIAA levels across the groups. However, the means of 5-HIAA levels were marginally lower in the HIV-only and HIV/TB co-infected groups compared to the healthy controls. This pattern is visually depicted in a box and whiskers plot (Fig. 2), which illustrates the distribution of 5-HIAA levels across the cohorts.

## Discussion

### HIV and its metabolic consequences on amino acids

In the HIV cohort, most amino acids were decreased compared to healthy controls, suggesting potential gastrointestinal (GI) malabsorption linked to HIV-associated enteropathy (Anton et al., 2000; Poles et al., 2001). HIV disrupts the GI mucosal epithelial barrier, leading to systemic immune activation and inflammation similar to inflammatory bowel diseases (Brenchley et al., 2006; Caradonna et al., 2000; Nazli et al., 2010).

A study using a ^15^N glycine stable isotope showed an anabolic protein turnover in HIV-only patients but no significant alterations in protein flux, degradation, or synthesis in TB-only and HIV/TB co-infected states (Paton et al., 2003), suggesting that HIV-related metabolic disruptions might be less pronounced in co-infected individuals. Inflammatory conditions like colitis, which share some pathophysiological features with HIV-associated GI complications, show that chronic inflammation can increase protein synthesis in the colon, spleen, and ileum while decreasing it in skeletal muscle. This reflects a shift in metabolism to support vital organs at the expense of muscle protein reserves (Mercier et al., 2002).

The significant reduction in tryptophan, isoleucine, and glutamic acid likely reflect their increased use in gluconeogenesis and the tricarboxylic acid (TCA) cycle under HIV stress, aiding glucose homeostasis (Namikawa-Kanai et al., 2020; Pasini et al., 2018). The reduced glutamine/glutamic acid ratio suggests enhanced glutaminase activity to support TCA cycle functions under energy stress (Mazat & Ransac, 2019).

Ethanolamine and phosphoethanolamine, used for cellular and mitochondrial phospholipid biosynthesis, were reduced (St Germain et al., 2023; Vance, 2015), potentially affecting mitochondrial function and highlighting the interplay between HIV infection and mitochondrial dysfunction.

### TB-driven changes in amino acid profiles

In TB subjects, 27 out of 37 analysed amino acids were elevated compared to healthy controls, suggesting impaired protein synthesis and enhanced catabolism potentially leading to increased amino acid oxidation (Zhou et al., 2013). Significantly elevated amino acids such as kynurenine, hydroxykynurenine, leucine, isoleucine, α-aminoadipic acid, arginine, and asparagine indicate increased synthesis of TCA cycle intermediates from amino acids used for oxidative phosphorylation (Leandro & Houten, 2020; Namikawa-Kanai et al., 2020; Pasini et al., 2018). Non-proteinogenic amino acids like kynurenine, carnosine, and α-aminoadipic acid, synthesised from their proteinogenic precursors, highlight an altered metabolic state due to the infection (Derave et al., 2010; Sacksteder et al., 2000; Savitz, 2020).

Increased saccharopine and α-aminoadipic acid levels, along with the saccharopine/lysine ratio, indicate enhanced saccharopine pathway activity. This likely represents an adaptive response to *Mtb* infection, optimising lysine use for NADH and FADH generation through the oxidation of glutaryl-CoA, despite consuming α-ketoglutarate, a TCA cycle intermediate (Leandro & Houten, 2020; Sacksteder et al., 2000). Increased kynurenine, hydroxykynurenine, and kynurenine/tryptophan ratio suggest heightened kynurenine pathway activity, possibly due to infection-induced proteolysis or *Mtb*’s ability to synthesise tryptophan de novo (Gautam et al., 2018). The elevated branched-chain amino acids (BCAAs) and increased carnosine levels support the notion of heightened proteolysis in TB, particularly from skeletal muscle. This process releases amino acids for energy production and gluconeogenesis but also cause BCAA accumulation in muscle tissue due to inhibited catabolism under conditions like cachexia or starvation (Derave et al., 2010; Holeček, 2020).

The decrease in urinary 3-methylhistidine levels contrasts with its typical increase associated with muscle wasting and cachexia—characterised by significant unintentional weight loss—commonly observed in TB patients (Luies & Du Preez, 2020). This discrepancy may be influenced by dietary variations, particularly lower read meat consumption in rural communities (Cross et al., 2011; Kochlik et al., 2018), and could also be exacerbated by the loss of appetite in TB patients (Luies & Du Preez, 2020). The significant decrease in glycine levels could indicate diabetes-associated hyperglycaemia, a known phenomenon in obesity and diabetes (Adeva-Andany et al., 2018; Takashina et al., 2016). TB has been linked to stress-induced hyperglycaemia in up to 87% of infected patients without pre-existing diabetes (Magee et al., 2018; Ngo et al., 2021). This relationship is particularly relevant in regions like South Africa where high diabetes prevalence and limited diagnostic resources complicate disease management (Olivier & Luies, 2023; Pillay & Aldous, 2016). To this end, the urine metabolome of TB patients shows significant amino acid alterations indicative of type II diabetes and/or insulin resistance, including the previously mentioned decreased glycine and increased BCAAs and α-aminoadipic acid. Insulin resistance, known to induce skeletal muscle wasting via the ubiquitin proteasome proteolytic pathway, underscores the complex metabolic disruptions in TB (Price et al., 1996; Wang et al., 2006). Similar amino acid changes resembling diabetes have been reported in South African TB patients (Luies & Loots, 2016).

### Acylcarnitines dynamics in TB

Although no acylcarnitines showed significant changes compared to the healthy control group due to high FDR values, a trend of elevated medium-chain acylcarnitines was observed in the TB-only group. Medium-chain acylcarnitine levels were consistently increased, with *p*-values < 0.05 for C6 and < 0.1 for others. This elevation may be linked to increased fatty acid oxidation associated with wasting/cachexia, as previously observed TB-positive cohorts with depleted fatty acids and free carnitine levels alongside increased medium-chain acylcarnitines (Che et al., 2018; Fukawa et al., 2016; Vrieling et al., 2018). In contrast to medium-chain acylcarnitines, which are exclusively produced by fatty acid metabolism, short-chain acylcarnitines are also formed from glucose and amino acids (Makrecka-Kuka et al., 2017). This may explain why short-chain acylcarnitines remained relatively unaffected in the TB-only and HIV/TB co-infected cohorts, which are expected to be in a glucose-depleted state with increased fatty acid oxidation for energy. Additionally, medium-chain acylcarnitines have been shown to downregulate mitochondrial complex V in type II diabetes, reducing mitochondrial oxidative capacity (Batchuluun et al., 2018). Thus, medium-chain acylcarnitines might also contribute to a diabetic-like profile in this cohort.

### Intersecting metabolic pathways in HIV/TB co-infection

Unlike the clear trends seen in HIV and TB groups, the HIV/TB co-infected cohort did not show a distinct pattern in amino acid levels compared to healthy controls. The HIV group had decreased amino acids indicative of anabolic protein turnover, while the TB group had increased levels suggestive of catabolic protein turnover. The co-infected group’s mixed profile may reflect a balance between these effects, possibly due to the interaction between HIV and *Mtb* infections. This interplay might neutralise the distinct metabolic impacts seen in mono-infections. Paton et al. (2003) observed that the heightened protein anabolism seen in HIV was absent in HIV/TB co-infection, suggesting that *Mtb* may modulate HIV-related metabolic responses.

Metabolic alterations in the co-infected group aligned with either HIV or TB profiles, indicating similar metabolic drivers. Fewer significant metabolic changes in the co-infected group (*n* = 4) compared to the HIV (*n* = 7) and TB (*n* = 11) underscore a lack of pronounced anabolic or catabolic shifts in protein metabolism.

The HIV/TB co-infected cohort’s profile was more influenced by TB, with shared features like increased hydroxykynurenine, saccharopine, a higher saccharopine/lysine ratio, and decreased glycine. Elevated medium-chain acylcarnitines in this group mirrored those seen in the TB cohort, hinting at increased fatty acid oxidation possibly linked to dysfunctional mitochondrial respiration often associated with diabetes. Decreased tryptophan levels in the co-infected group might stem from HIV-induced malabsorption and *Mtb*-related kynurenine pathway activation, though not as pronounced as in the TB-only group. This complex interaction affects essential metabolic pathways (Herbert et al., 2023), like the saccharopine pathway, involved in lysine degradation and potentially contributing to metabolic adaptations required for oxidative phosphorylation in co-infected individuals.

**Fig. 3:**
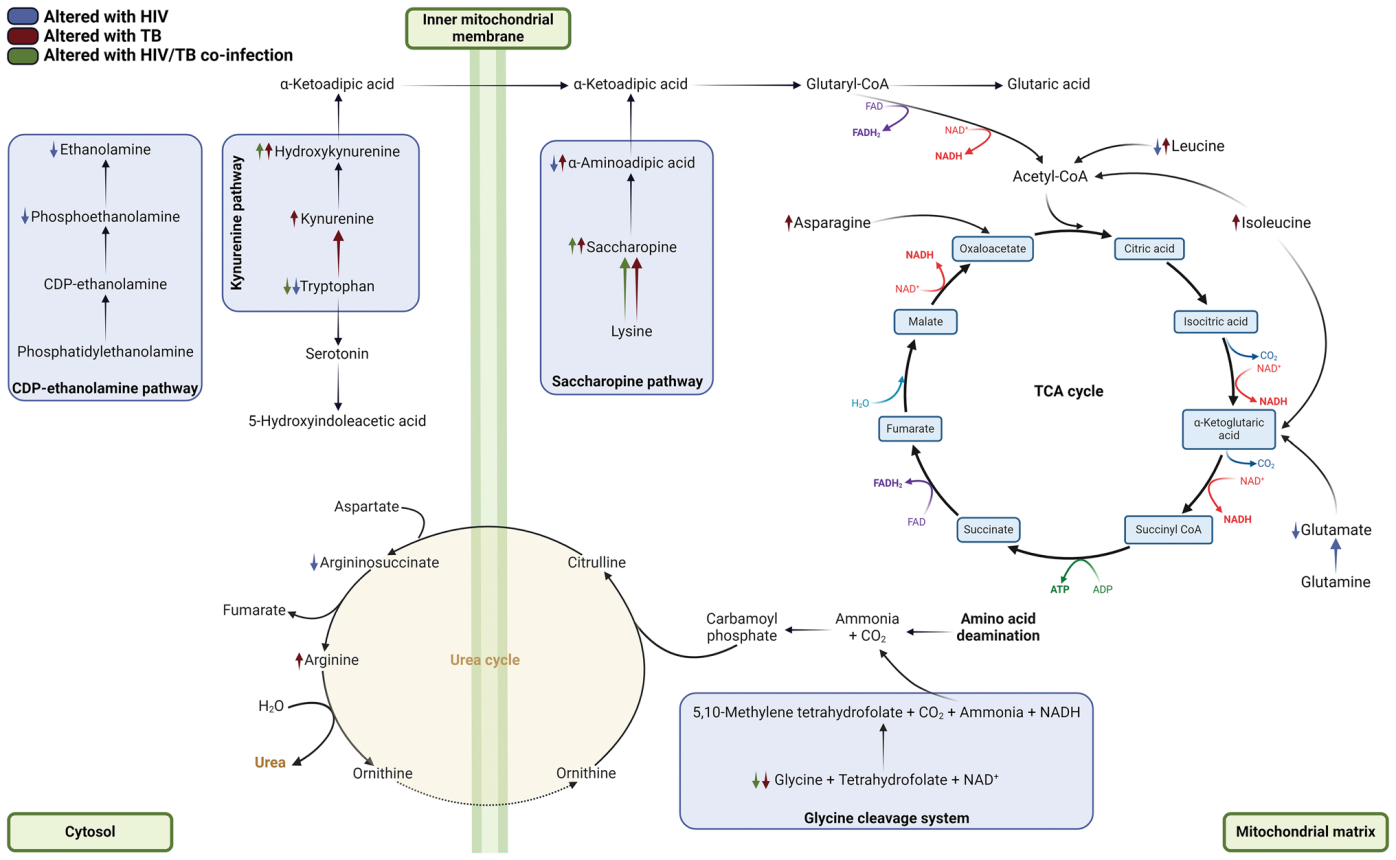
Amino acid metabolism pathways influenced by infection states. This figure illustrates how HIV, TB, and HIV/TB co-infection affect amino acid metabolic pathways, using colour-coded arrows to indicate alterations, providing insight into the metabolic disturbances associated with these disease states. CDP-ethanolamine, cytidine diphosphate-ethanolamine; CO_2_, carbon dioxide; TCA cycle, tricarboxylic acid cycle; NAD^+^, nicotinamide adenine dinucleotide; FAD, flavin adenine dinucleotide [Created using BioRender]

Metabolic trends also show a correspondence between tryptophan and 5-HIAA levels across the infection states, with decreased levels in HIV and co-infection and increased levels in TB, indicating consistent responses to these infections. This highlights the intricate metabolic interactions in HIV/TB co-infection, as summarised in Fig. 3.

### Metabolic insights and health implications from amino acid and acylcarnitine disturbances

Disruptions in amino acid levels impact protein synthesis, immune function, and energy processes (Wu, 2010, 2013), exacerbating wasting and cachexia in HIV and TB patients (Luies & Du Preez, 2020). Lower amino acid levels in HIV may activate enzymes that inhibit RNA virus replication, potentially affecting HIV severity (Afroz et al., 2020; Del Pino et al., 2012). In HIV/TB co-infection, elevated kynurenine pathway activity could compromise immune response effectiveness (Mbongue et al., 2015).

Amino acids are crucial for neurotransmitter synthesis, affecting neurological health, which is especially concerning in HIV due to potential neurotoxic effects (Ling et al., 2023). While our study did not find significant changes in systemic serotonin metabolic pathways (via 5-HIAA), the depletion of tryptophan in HIV-related infections could still disrupt serotonin production within the brain, which synthesises its own serotonin independently from peripheral sources (Ogawa et al., 2023).

Elevated medium-chain acylcarnitines in TB and HIV/TB co-infected patients suggest heightened fatty acid oxidation, potentially a response to chronic infection and inflammation (Che et al., 2018; Li et al., 2018). However, sustained changes in acylcarnitine metabolism could disrupt mitochondrial function and energy homeostasis, contributing to muscle wasting, fatigue, reduced physiological resilience, and conditions like insulin resistance and diabetes (Batchuluun et al., 2018; Kuratsune et al., 1994; Malaguarnera et al., 2006).

Addressing these metabolic alterations is crucial for comprehensive disease management in HIV, TB, and co-infected patients, aiming not just to control infection but also to mitigate its systemic and metabolic consequences.

### Limitations

The findings of this investigation offer significant insights into the metabolic changes in individuals with HIV/TB co-infection, highlighting the complex dynamics between these diseases. However, the study has limitations, particularly regarding the applicability of our findings. Despite efforts to align demographic factors and employ rigorous selection criteria, the small sample size limits our ability to control for all potential confounders and reduces the statistical power of our observations. Furthermore, the absence of complete CD4 cell count and viral load data across all patient groups restricts our ability to fully analyse their potential impact on metabolic status and amino acid or acylcarnitine differences among co-infected patients. Moreover, focusing on a specific geographical area and reducing variability in *Mtb* strains may limit the generalisability of our findings to broader populations with diverse *Mtb* strains. Future studies should prioritise gathering supplementary clinical data, such as BMI, to enhance our understanding of the metabolic changes associated with HIV/TB co-infection. Larger-scale studies with stringent matching criteria are essential to validate these findings and to further unravel the metabolic interactions between HIV and TB.

## Conclusion

This study’s comprehensive analysis of urinary acylcarnitine and amino acid profiles in patients with HIV/TB co-infection has illuminated the significant metabolic disturbances associated with this complex syndemic. Our findings highlight the critical role of TB in driving these metabolic changes, marked by notable alterations in both amino acid and acylcarnitine profiles.

The observed increase in medium-chain acylcarnitines suggests enhanced fatty acid oxidation, a hallmark of cachexia prevalent in TB, contributing significantly to muscle wasting and severe weight loss. Additionally, the altered amino acid levels indicate disruptions in glucose metabolism, suggesting a trend towards diabetes-like symptoms in both TB and co-infected groups. This finding is particularly critical as it highlights a potential intersection of metabolic and infectious disease processes, where TB-associated metabolic changes could either exacerbate or mask underlying conditions such as diabetes.

Recognising TB as the predominant driver of these metabolic disruptions in co-infected patients provides essential insights into disease progression and treatment response. Effectively managing these metabolic changes through targeted therapeutic strategies could substantially enhance clinical outcomes. However, it is important to note that the small sample size in our study limits the statistical power of these observations.

Further research should focus on validating these findings in larger and more diverse populations to clarify the mechanisms driving these metabolic changes. Advancing our understanding of the metabolic impacts of HIV/TB co-infection will enable tailored interventions to address not only the infections but also their profound systemic effects, ultimately improving patient health and quality of life.

## Supplementary Information

Below is the link to the electronic supplementary material.

Supplementary file1 (PDF 572 KB)

## Data Availability

All data that form part of this paper and its supplementary material are free to obtain. Datasets are available on Biostudies, using Accession Number S-BSST1402.

## References

[CR1] Adeva-Andany, M., Souto-Adeva, G., Ameneiros-Rodríguez, E., Fernández-Fernández, C., Donapetry-García, C., & Domínguez-Montero, A. (2018). Insulin resistance and glycine metabolism in humans. *Amino Acids,**50*, 11–27. 29094215 10.1007/s00726-017-2508-0

[CR2] Afroz, S., Battu, S., Giddaluru, J., & Khan, N. (2020). Dengue virus induced COX-2 signaling is regulated through nutrient sensor GCN2. *Frontiers in Immunology,**11*, 1831. 32903536 10.3389/fimmu.2020.01831 PMC7438581

[CR3] Anton, P. A., Elliott, J., Poles, M. A., McGowan, I. M., Matud, J., Hultin, L. E., Grovit-Ferbas, K., Mackay, C. R., Chen, I. S., & Giorgi, J. V. (2000). Enhanced levels of functional HIV-1 co-receptors on human mucosal T cells demonstrated using intestinal biopsy tissue. *AIDS,**14*, 1761–1765. 10985313 10.1097/00002030-200008180-00011

[CR4] Batchuluun, B., Al Rijjal, D., Prentice, K. J., Eversley, J. A., Burdett, E., Mohan, H., Bhattacharjee, A., Gunderson, E. P., Liu, Y., & Wheeler, M. B. (2018). Elevated medium-chain acylcarnitines are associated with gestational diabetes mellitus and early progression to type 2 diabetes and induce pancreatic β-cell dysfunction. *Diabetes,**67*, 885–897. 29436377 10.2337/db17-1150 PMC5910003

[CR5] Bi, H., Guo, Z., Jia, X., Liu, H., Ma, L., & Xue, L. (2020). The key points in the pre-analytical procedures of blood and urine samples in metabolomics studies. *Metabolomics,**16*, 1–15. 10.1007/s11306-020-01666-2 32451742

[CR6] Brenchley, J. M., Price, D. A., Schacker, T. W., Asher, T. E., Silvestri, G., Rao, S., Kazzaz, Z., Bornstein, E., Lambotte, O., & Altmann, D. (2006). Microbial translocation is a cause of systemic immune activation in chronic HIV infection. *Nature Medicine,**12*, 1365–1371. 17115046 10.1038/nm1511

[CR7] Caradonna, L., Amati, L., Magrone, T., Pellegrino, N., Jirillo, E., & Caccavo, D. (2000). Invited review: Enteric bacteria, lipopolysaccharides and related cytokines in inflammatory bowel disease: Biological and clinical significance. *Journal of Endotoxin Research,**6*, 205–214. 11052175

[CR8] Che, N., Ma, Y., Ruan, H., Xu, L., Wang, X., Yang, X., & Liu, X. (2018). Integrated semi-targeted metabolomics analysis reveals distinct metabolic dysregulation in pleural effusion caused by tuberculosis and malignancy. *Clinica Chimica Acta,**477*, 81–88. 10.1016/j.cca.2017.12.003 29208371

[CR9] Cho, Y., Park, Y., Sim, B., Kim, J., Lee, H., Cho, S.-N., Kang, Y., & Lee, S.-G. (2020). Identification of serum biomarkers for active pulmonary tuberculosis using a targeted metabolomics approach. *Scientific Reports,**10*, 1–11. 32123207 10.1038/s41598-020-60669-0 PMC7052258

[CR10] Clark, Z. D., Cutler, J. M., & Frank, E. L. (2017). Practical LC-MS/MS method for 5-hydroxyindoleacetic acid in urine. *The Journal of Applied Laboratory Medicine,**1*, 387–399. 33636811 10.1373/jalm.2016.021675

[CR11] Cross, A. J., Major, J. M., & Sinha, R. (2011). Urinary biomarkers of meat consumption. *Cancer Epidemiology, Biomarkers & Prevention,**20*, 1107–1111. 10.1158/1055-9965.EPI-11-0048 PMC3111815 21527577

[CR45] Del Pino, J., Jiménez, J.L., Ventoso, I., Castello, A., Muñoz-Fernández, M.Á., de Haro, C., & Berlanga, J. J. (2012). GCN2 has inhibitory effect on human immunodeficiency virus-1 protein synthesis and is cleaved upon viral infection. *PLoS One*, e47272. 10.1371/journal.pone.0047272 10.1371/journal.pone.0047272 PMC3479103 23110064

[CR12] Derave, W., Everaert, I., Beeckman, S., & Baguet, A. (2010). Muscle carnosine metabolism and β-alanine supplementation in relation to exercise and training. *Sports Medicine,**40*, 247–263. 20199122 10.2165/11530310-000000000-00000

[CR13] Eriksson, L., Byrne, T., Johansson, E., Trygg, J., & Vikström, C. (2013) *Multi-and megavariate data analysis basic principles and applications*. Umetrics Academy.

[CR14] Food and Drug Administration. (2018). *Bioanalytical method validation. Guidance for industry*. US Department of Health and Human Services. Center for Drug Evaluation and Research (CDER), Center for Veterinary Medicine (CVM), Silver Spring, MD

[CR15] Fukawa, T., Yan-Jiang, B. C., Min-Wen, J. C., Jun-Hao, E. T., Huang, D., Qian, C.-N., Ong, P., Li, Z., Chen, S., & Mak, S. Y. (2016). Excessive fatty acid oxidation induces muscle atrophy in cancer cachexia. *Nature Medicine,**22*, 666–671. 27135739 10.1038/nm.4093

[CR16] Gautam, U. S., Foreman, T. W., Bucsan, A. N., Veatch, A. V., Alvarez, X., Adekambi, T., Golden, N. A., Gentry, K. M., Doyle-Meyers, L. A., & Russell-Lodrigue, K. E. (2018). In vivo inhibition of tryptophan catabolism reorganizes the tuberculoma and augments immune-mediated control of Mycobacterium tuberculosis. *Proceedings of the National Academy of Sciences,**115*, E62–E71. 10.1073/pnas.1711373114 PMC5776797 29255022

[CR17] Herbert, C., Luies, L., Loots, D. T., & Williams, A. A. (2023). The metabolic consequences of HIV/TB co-infection. *Bmc Infectious Diseases,**23*, 536. 37592227 10.1186/s12879-023-08505-4 PMC10436461

[CR18] Holeček, M. (2020). Why are branched-chain amino acids increased in starvation and diabetes? *Nutrients,**12*, 3087. 33050579 10.3390/nu12103087 PMC7600358

[CR19] Joint United Nations Programme on HIV/AIDS (2023) Global HIV&AIDS Statistics-fact sheet 2023.

[CR20] Kochlik, B., Gerbracht, C., Grune, T., & Weber, D. (2018). The influence of dietary habits and meat consumption on plasma 3-methylhistidine—A potential marker for muscle protein turnover. *Molecular Nutrition & Food Research,**62*, 1701062. 29573154 10.1002/mnfr.201701062 PMC5969234

[CR21] Kuratsune, H., Yamaguti, K., Takahashi, M., Misaki, H., Tagawa, S., & Kitani, T. (1994). Acylcarnitine deficiency in chronic fatigue syndrome. *Clinical Infectious Diseases,**18*, S62–S67. 8148455 10.1093/clinids/18.supplement_1.s62

[CR22] Leandro, J., & Houten, S. M. (2020). The lysine degradation pathway: Subcellular compartmentalization and enzyme deficiencies. *Molecular Genetics and Metabolism,**131*, 14–22. 32768327 10.1016/j.ymgme.2020.07.010

[CR23] Li, X., Wu, T., Jiang, Y., Zhang, Z., Han, X., Geng, W., Ding, H., Kang, J., Wang, Q., & Shang, H. (2018). Plasma metabolic changes in Chinese HIV-infected patients receiving lopinavir/ritonavir based treatment: Implications for HIV precision therapy. *Cytokine,**110*, 204–212. 29778008 10.1016/j.cyto.2018.05.001

[CR24] Liebenberg, C., Luies, L., & Williams, A. A. (2021). Metabolomics as a tool to investigate HIV/TB co-infection. *Frontiers in Molecular Biosciences*, *8*, 692823· 10.3389/fmolb.2021.692823 10.3389/fmolb.2021.692823 PMC8565463 34746228

[CR25] Ling, Z.-N., Jiang, Y.-F., Ru, J.-N., Lu, J.-H., Ding, B., & Wu, J. (2023). Amino acid metabolism in health and disease. *Signal Transduction and Targeted Therapy,**8*, 345. 37699892 10.1038/s41392-023-01569-3 PMC10497558

[CR26] Luies, L., & Du Preez, I. (2020). The echo of pulmonary tuberculosis: Mechanisms of clinical symptoms and other disease-induced systemic complications. *Clinical Microbiology Reviews,**33*, e00036-e120. 32611585 10.1128/CMR.00036-20 PMC7331478

[CR27] Luies, L., & Loots, D. (2016). Tuberculosis metabolomics reveals adaptations of man and microbe in order to outcompete and survive. *Metabolomics,**12*, 1–9.

[CR28] Magee, M. J., Salindri, A. D., Kyaw, N. T. T., Auld, S. C., Haw, J. S., & Umpierrez, G. E. (2018). Stress hyperglycemia in patients with tuberculosis disease: Epidemiology and clinical implications. *Current Diabetes Reports,**18*, 1–10. 30090969 10.1007/s11892-018-1036-y PMC6309553

[CR29] Makrecka-Kuka, M., Sevostjanovs, E., Vilks, K., Volska, K., Antone, U., Kuka, J., Makarova, E., Pugovics, O., Dambrova, M., & Liepinsh, E. (2017). Plasma acylcarnitine concentrations reflect the acylcarnitine profile in cardiac tissues. *Scientific Reports,**7*, 17528. 29235526 10.1038/s41598-017-17797-x PMC5727517

[CR30] Malaguarnera, M., Risino, C., Gargante, M. P., Oreste, G., Barone, G., Tomasello, A. V., Costanzo, M., & Cannizzaro, M. A. (2006). Decrease of serum carnitine levels in patients with or without gastrointestinal cancer cachexia. *World Journal of Gastroenterology: WJG,**12*, 4541. 16874868 10.3748/wjg.v12.i28.4541 PMC4125643

[CR31] Mazat, J.-P., & Ransac, S. (2019). The fate of glutamine in human metabolism. The interplay with glucose in proliferating cells. *Metabolites,**9*, 81. 31027329 10.3390/metabo9050081 PMC6571637

[CR32] Mbongue, J. C., Nicholas, D. A., Torrez, T. W., Kim, N.-S., Firek, A. F., & Langridge, W. H. (2015). The role of indoleamine 2, 3-dioxygenase in immune suppression and autoimmunity. *Vaccines,**3*, 703–729. 26378585 10.3390/vaccines3030703 PMC4586474

[CR33] Melone, M. A. B., Valentino, A., Margarucci, S., Galderisi, U., Giordano, A., & Peluso, G. (2018). The carnitine system and cancer metabolic plasticity. *Cell Death & Disease,**9*, 1–12. 29445084 10.1038/s41419-018-0313-7 PMC5833840

[CR34] Mercier, S., Breuille, D., Mosoni, L., Obled, C., & Patureau Mirand, P. (2002). Chronic inflammation alters protein metabolism in several organs of adult rats. *The Journal of Nutrition,**132*, 1921–1928. 12097671 10.1093/jn/132.7.1921

[CR35] Munn, D. H., & Mellor, A. L. (2013). Indoleamine 2, 3 dioxygenase and metabolic control of immune responses. *Trends in Immunology,**34*, 137–143. 23103127 10.1016/j.it.2012.10.001 PMC3594632

[CR36] Namikawa-Kanai, H., Miyazaki, T., Matsubara, T., Shigefuku, S., Ono, S., Nakajima, E., Morishita, Y., Honda, A., Furukawa, K., & Ikeda, N. (2020). Comparison of the amino acid profile between the nontumor and tumor regions in patients with lung cancer. *American Journal of Cancer Research,**10*, 2145. 32775007 PMC7407354

[CR37] Nazli, A., Chan, O., Dobson-Belaire, W. N., Ouellet, M., Tremblay, M. J., Gray-Owen, S. D., Arsenault, A. L., & Kaushic, C. (2010). Exposure to HIV-1 directly impairs mucosal epithelial barrier integrity allowing microbial translocation. *PLoS Pathogens,**6*, e1000852. 20386714 10.1371/journal.ppat.1000852 PMC2851733

[CR38] Ngo, M. D., Bartlett, S., & Ronacher, K. (2021). Diabetes-associated susceptibility to tuberculosis: Contribution of hyperglycemia vs. *Dyslipidemia. Microorganisms,**9*, 2282. 34835407 10.3390/microorganisms9112282 PMC8620310

[CR39] Ogawa, M., Shimizu, F., Ishii, Y., Takao, T., & Takada, A. (2023). Uniqueness of tryptophan in the transport system in the brain and peripheral tissues. *Food and Nutrition Sciences,**14*, 401–414.

[CR40] Olivier, C., & Luies, L. (2023). WHO goals and beyond: Managing HIV/TB co-infection in South Africa. *SN Comprehensive Clinical Medicine,**5*, 251.

[CR41] Pasini, E., Corsetti, G., Aquilani, R., Romano, C., Picca, A., Calvani, R., & Dioguardi, F. S. (2018). Protein-amino acid metabolism disarrangements: The hidden enemy of chronic age-related conditions. *Nutrients,**10*, 391. 29565819 10.3390/nu10040391 PMC5946176

[CR42] Paton, N. I., Ng, Y.-M., Chee, C. B., Persaud, C., & Jackson, A. A. (2003). Effects of tuberculosis and HIV infection on whole-body protein metabolism during feeding, measured by the [15N] glycine method. *The American Journal of Clinical Nutrition,**78*, 319–325. 12885716 10.1093/ajcn/78.2.319

[CR43] Peltenburg, N. C., Schoeman, J. C., Hou, J., Mora, F., Harms, A. C., Lowe, S. H., Bierau, J., Bakker, J. A., Verbon, A., & Hankemeier, T. (2018). Persistent metabolic changes in HIV-infected patients during the first year of combination antiretroviral therapy. *Scientific Reports,**8*, 16947. 30446683 10.1038/s41598-018-35271-0 PMC6240055

[CR44] Pillay, S., & Aldous, C. (2016). Introducing a multifaceted approach to the management of diabetes mellitus in resource-limited settings. *SAMJ: South African Medical Journal,**106*, 456–458. 10.7196/SAMJ.2016.v106i5.10409 27138660

[CR46] Poles, M. A., Elliott, J., Taing, P., Anton, P. A., & Chen, I. S. (2001). A preponderance of CCR5+ CXCR4+ mononuclear cells enhances gastrointestinal mucosal susceptibility to human immunodeficiency virus type 1 infection. *Journal of Virology,**75*, 8390–8399. 11507184 10.1128/JVI.75.18.8390-8399.2001 PMC115084

[CR47] Price, S. R., Bailey, J. L., Wang, X., Jurkovitz, C., England, B. K., Ding, X., Phillips, L. S., & Mitch, W. E. (1996). Muscle wasting in insulinopenic rats results from activation of the ATP-dependent, ubiquitin-proteasome proteolytic pathway by a mechanism including gene transcription. *The Journal of Clinical Investigation,**98*, 1703–1708. 8878419 10.1172/JCI118968 PMC507607

[CR48] Qu, Q., Zeng, F., Liu, X., Wang, Q., & Deng, F. (2016). Fatty acid oxidation and carnitine palmitoyltransferase I: Emerging therapeutic targets in cancer. *Cell Death & Disease,**7*, e2226–e2226. 27195673 10.1038/cddis.2016.132 PMC4917665

[CR49] Roth, W., Zadeh, K., Vekariya, R., Ge, Y., & Mohamadzadeh, M. (2021). Tryptophan metabolism and gut-brain homeostasis. *International Journal of Molecular Sciences,**22*, 2973. 33804088 10.3390/ijms22062973 PMC8000752

[CR50] Sacksteder, K. A., Biery, B. J., Morrell, J. C., Goodman, B. K., Geisbrecht, B. V., Cox, R. P., Gould, S. J., & Geraghty, M. T. (2000). Identification of the α-aminoadipic semialdehyde synthase gene, which is defective in familial hyperlysinemia. *The American Journal of Human Genetics,**66*, 1736–1743. 10775527 10.1086/302919 PMC1378037

[CR51] Saharia, K. K., & Koup, R. A. (2013). T cell susceptibility to HIV influences outcome of opportunistic infections. *Cell,**155*, 505–514. 24243010 10.1016/j.cell.2013.09.045 PMC3858849

[CR52] Savitz, J. (2020). The kynurenine pathway: A finger in every pie. *Molecular Psychiatry,**25*, 131–147. 30980044 10.1038/s41380-019-0414-4 PMC6790159

[CR53] Schooneman, M. G., Vaz, F. M., Houten, S. M., & Soeters, M. R. (2013). Acylcarnitines: Reflecting or inflicting insulin resistance? *Diabetes,**62*, 1–8. 23258903 10.2337/db12-0466 PMC3526046

[CR54] Sitole, L. J., Tugizimana, F., & Meyer, D. (2019). Multi-platform metabonomics unravel amino acids as markers of HIV/combination antiretroviral therapy-induced oxidative stress. *Journal of Pharmaceutical and Biomedical Analysis,**176*, 112796. 31398507 10.1016/j.jpba.2019.112796

[CR55] Smith, E. H., & Matern, D. (2010). Acylcarnitine analysis by tandem mass spectrometry. *Current Protocols in Human Genetics,**64*, 17.8.1-17.8.20. 10.1002/0471142905.hg1708s64 20063265

[CR56] St Germain, M., Iraji, R., & Bakovic, M. (2023). Phosphatidylethanolamine homeostasis under conditions of impaired CDP-ethanolamine pathway or phosphatidylserine decarboxylation. *Frontiers in Nutrition,**9*, 1094273. 36687696 10.3389/fnut.2022.1094273 PMC9849821

[CR57] Takashina, C., Tsujino, I., Watanabe, T., Sakaue, S., Ikeda, D., Yamada, A., Sato, T., Ohira, H., Otsuka, Y., & Oyama-Manabe, N. (2016). Associations among the plasma amino acid profile, obesity, and glucose metabolism in Japanese adults with normal glucose tolerance. *Nutrition & Metabolism,**13*, 1–10. 26788116 10.1186/s12986-015-0059-5 PMC4717594

[CR58] Tong, T., & Zhao, H. (2008). Practical guidelines for assessing power and false discovery rate for a fixed sample size in microarray experiments. *Statistics in Medicine,**27*, 1960–1972. 18338314 10.1002/sim.3237 PMC3157366

[CR59] Vance, J. E. (2015). Phospholipid synthesis and transport in mammalian cells. *Traffic,**16*, 1–18. 25243850 10.1111/tra.12230

[CR60] Vrieling, F., Ronacher, K., Kleynhans, L., Van Den Akker, E., Walzl, G., Ottenhoff, T. H., & Joosten, S. A. (2018). Patients with concurrent tuberculosis and diabetes have a pro-atherogenic plasma lipid profile. *eBioMedicine,**32*, 192–200. 29779698 10.1016/j.ebiom.2018.05.011 PMC6020709

[CR61] Wang, X., Hu, Z., Hu, J., Du, J., & Mitch, W. E. (2006). Insulin resistance accelerates muscle protein degradation: Activation of the ubiquitin-proteasome pathway by defects in muscle cell signaling. *Endocrinology,**147*, 4160–4168. 16777975 10.1210/en.2006-0251

[CR62] Weiner, J., Parida, S. K., Maertzdorf, J., Black, G. F., Repsilber, D., Telaar, A., Mohney, R. P., Arndt-Sullivan, C., Ganoza, C. A., & Fae, K. C. (2012). Biomarkers of inflammation, immunosuppression and stress are revealed by metabolomic profiling of tuberculosis patients. *PLoS ONE,**7*, e40221. 22844400 10.1371/journal.pone.0040221 PMC3402490

[CR63] World Health Organization (2023) Global tuberculosis report 2023

[CR64] Wu, G. (2010). Functional amino acids in growth, reproduction, and health. *Advances in Nutrition,**1*, 31–37. 22043449 10.3945/an.110.1008 PMC3042786

[CR65] Wu, G. (2013). Functional amino acids in nutrition and health. *Amino Acids,**45*, 407–411. 23595206 10.1007/s00726-013-1500-6

[CR66] Zhou, A., Ni, J., Xu, Z., Wang, Y., Lu, S., Sha, W., Karakousis, P. C., & Yao, Y.-F. (2013). Application of 1H NMR spectroscopy-based metabolomics to sera of tuberculosis patients. *Journal of Proteome Research,**12*, 4642–4649. 23980697 10.1021/pr4007359 PMC3838786

