# Supplementary material for: Characterising the urinary acylcarnitine and amino acid profiles of HIV/TB co-infection, using LC–MS metabolomics
Source: Metabolomics. 2024 Aug 3;20(5):92. doi: 10.1007/s11306-024-02161-8 (PMC11297823; doi:10.1007/s11306-024-02161-8)
Supplement: Supplementary file 1 — Supplementary file1 (PDF 572 KB) [file 11306_2024_2161_MOESM1_ESM.pdf]

SUPPLEMENTARY MATERIAL: Characterising the urinary acylcarnitine and amino acid profiles of HIV/TB co-infection, using LC-MS metabolomics

Table S1: Acylcarnitine isotope working solution prepared in H_2O

Acylcarnitine isotope	Concentration (µmol/L)
Acetyl-L-carnitine-d3 HCl	0.3
Propionyl-L-carnitine-d3 HCl	0.3
Isovaleryl-L-carnitine-d9 HCl	0.3
Hexanoyl-L-carnitine-d3 HCl	0.3
Octanoyl-L-carnitine-d3 HCl	0.3
Decanoyl-L-carnitine-d3 HCl	0.3
Dodecanoyl-L-carnitine-d3 HCl	0.3

Table S2: HPLC-MS/MS chromatography gradient elution conditions for acylcarnitine and 5-HIAA analyses

Acylcarnitine analysis			5-HIAA analysis		
**Time (min)**	**ACN (%)**	**FR (mL/min)**	**Time (min)**	**Methanol (%)**	**FR (mL/min)**
**0**	5	0.3	**0**	15	0.3
**15**	25	0.3	**6**	33	0.3
**17**	25	0.3	**11**	100	0.3
**18**	30	0.3	**13**	100	0.3
**19**	30	0.3			
**28.5**	64.3	0.3			
**31**	100	0.5			
**32**	100	0.5			

Table S3: Mass spectrometer source parameters for acylcarnitine and 5-HIAA analyses

Method	Acylcarnitine analysis	5-HIAA analysis
**Gas temperature (˚C)**	280	150
**Gas flow (L/minute)**	9	8
**Nebulizer (psi)**	45	20
**Sheath gas temperature (˚C)**	400	300
**Sheath gas flow (L/minute)**	12	12
**Capillary voltage (V)**	4 500	3 000
**Nozzle voltage (V)**	500	0

Table S4: MRM/dMRM transitions for acylcarnitines and 5-HIAA analyses

Analyte	Precursor ion (m/z)	Product ion (m/z)	Fragmentor voltage (V)	Collision energy (V)
**C2**	260.2	85.1	159	32
**C2 IS**	263.2	85.1	101	24
**C3**	274.2	85.1	111	24
**C3 IS**	277.2	85.1	107	26
**C5**	302.2	85.1	102	26
**C5 IS**	311.1	85.1	122	26
**C6**	316.2	85.1	121	24
**C6 IS**	319.3	85.1	117	30
**C8**	344.2	85.1	129	28
**C8 IS**	347.3	85.1	107	30
**C10**	372.3	85.1	144	32
**C10 IS**	375.3	85.1	117	30
**C12**	400.3	85.1	159	32
**C12 IS**	403.3	85.1	107	30
**5-HIAA**	192.1	146	80	16
**5-HIAA (qualifier)**	192.1	118.1	80	34
**5-HIAA IS**	195.17	148	80	18
**5-HIAA IS (qualifier)**	195.17	120.1	80	34

Abbreviations: IS: internal standard; C2: acetyl-L-carnitine; C3: propionyl-L-carnitine; C5: isovaleryl-L-carnitine; C6: hexanoyl-L-carnitine; C8: octanoyl-L-carnitine; C10: decanoyl-L-carnitine; C12: dodecanoyl-L-carnitine; 5-HIAA: 5-hydroxyindoleacetic acid.

Table S5: Calibrator concentrations used to construct calibration curves for acylcarnitines and 5-HIAA

Calibrator (µmol/L)	1	2	3	4	5	6	7	8	9	10
**C2**	0.05	0.1	0.2	0.3	0.75	1.5	3	4	12	25
**C3, C5**	0.005	0.015	0.05	0.1	0.2	0.3	0.750	1.5	3	4
**C6, C8, C10, C12**	0.0002	0.0004	0.0008	0.0024	0.005	0.0075	0.01	0.02	0.03	0.08
**5-HIAA**	0.1	0.25	0.5	3	8	12.5	25	50	75	100

Table S6: Creatinine-corrected concentrations for acylcarnitines and 5-HIAA, in (μmol/L)/(mmol/L) creatinine

ID	Group	C2	C3	C5	C6	C8	C10	C12	5-HIAA
**34**	HC	0.773	0.124	0.007	0.004	0.010	0.006	0.003	0.845
**36**	HC	1.511	0.577	0.057	0.004	0.016	0.005	0	0
**43**	HC	1.967	0	0	0	0	0	0	0
**44**	HC	0.622	0.166	0.006	0.001	0.004	0.004	0	1.073
**45**	HC	0	0	0	0	0	0	0	0
**47**	HC	0	0	0.003	0.004	0.006	0.007	0.003	1.351
**49**	HC	1.325	0.298	0.016	0.003	0.013	0.008	0.003	0.833
**59**	HC	0.933	0.339	0.034	0.003	0.011	0.008	0.004	1.237
**61**	HC	0.872	0.318	0.027	0.002	0.008	0.003	0.002	0.498
**64**	HC	1.549	0.372	0.057	0.005	0.024	0.011	0.003	1.184
**65**	HC	0.825	0.384	0.041	0.003	0.019	0.005	0.003	1.293
**67**	HC	0	0	0.003	0.001	0.002	0	0.003	1.009
**69**	HC	0.126	0	0.006	0.001	0.004	0.003	0	1.018
**116**	HC	2.196	0.209	0.070	0	0	0.003	0	0.380
**127**	HC	0.217	0.073	0.004	0.001	0.002	0.002	0	1.661
**147**	HC	0.233	0.105	0.022	0.001	0.004	0.004	0.001	1.092
**148**	HC	0.188	0.067	0.014	0.001	0.003	0.003	0	1.844
**155**	HC	0	0	0.009	0.005	0.007	0.010	0	1.573
**158**	HC	0.067	0	0.003	0.003	0.006	0.006	0.003	1.641
**159**	HC	1.072	0.290	0.022	0.006	0.019	0.013	0.002	1.143
**161**	HC	0	0	0	0	0	0	0	0
**162**	HC	0.581	0.084	0.010	0.006	0.013	0.016	0.005	1.239
**163**	HC	0.750	0.297	0.060	0	0.009	0	0	1.617
**165**	HC	0.590	0.159	0.016	0.003	0.008	0.006	0.002	1.330
**166**	HC	0	0	0	0	0.005	0	0.016	1.922
**167**	HC	0.397	0.097	0.019	0.001	0.006	0.005	0.004	2.579
**170**	HC	0.928	0.300	0.061	0.003	0.018	0.006	0.001	0.771
**171**	HC	0.604	0.130	0.006	0.001	0.004	0.003	0.002	1.085
**172**	HC	0.819	0.260	0.020	0.007	0.016	0.023	0.012	2.330
**173**	HC	0.867	0.149	0.011	0.003	0.012	0.007	0.003	2.230
**184**	HC	3.749	0.303	0.038	0.002	0.011	0.007	0.002	1.151
**185**	HC	1.397	0.198	0.029	0.003	0.015	0.013	0.004	0.946
**1**	TB-only	0.484	0.229	0.032	0.002	0.008	0.004	0.001	1.093
**3**	TB-only	1.065	0.358	0.027	0.007	0.022	0.011	0.002	1.695
**7**	TB-only	0.054	0.015	0.005	0.014	0.019	0.019	0.008	1.655

**12**	TB-only	0.175	0.051	0.006	0.001	0.002	0.002	0.002	0.751
**14**	TB-only	0.120	0.037	0.006	0.010	0.016	0.020	0.005	0.769
**19**	TB-only	0.211	0	0.004	0.012	0.018	0.031	0.012	1.527
**22**	TB-only	0.090	0.056	0.013	0.003	0.005	0.005	0.003	6.467
**23**	TB-only	0.556	0.221	0.021	0.005	0.016	0.014	0.004	0.540
**27**	TB-only	0.079	0.028	0.008	0.002	0.004	0.005	0.002	0.768
**90**	TB-only	0.692	0.185	0.006	0.002	0.006	0.004	0.001	0.871
**91**	TB-only	1.979	0.126	0.007	0.003	0.007	0.004	0.002	0.943
**123**	TB-only	0.353	0.064	0.003	0.003	0.007	0.007	0.003	0
**124**	TB-only	0	0	0.016	0.008	0.014	0.017	0.010	0
**128**	TB-only	0.022	0	0.002	0.003	0.005	0.006	0.002	0.510
**130**	TB-only	0.333	0.202	0.019	0.004	0.007	0.008	0.003	1.362
**132**	TB-only	0.402	0.057	0.006	0.003	0.007	0.006	0.004	1.960
**135**	TB-only	0	0	0.003	0.001	0.002	0.003	0.001	1.874
**140**	TB-only	0.191	0	0.028	0.002	0.003	0	0	1.139
**141**	TB-only	0.452	0.204	0.020	0.003	0.007	0	0.008	0.874
**144**	TB-only	1.935	0.645	0.040	0.003	0.010	0.005	0.001	1.843
**146**	TB-only	5.614	2.364	0.368	0.047	1.325	0.086	0.005	0.991
**152**	TB-only	0.297	0.069	0.006	0.003	0.005	0.004	0.002	0.916
**154**	TB-only	0.515	0	0	0	0.006	0	0	1.807
**157**	TB-only	0	0	0.005	0.001	0.002	0	0.003	2.106
**168**	TB-only	1.954	0.648	0.087	0.006	0.022	0.008	0.006	1.063
**169**	TB-only	0.692	0.272	0.029	0.002	0.009	0.004	0.002	1.377
**175**	TB-only	0.084	0	0.009	0.006	0.010	0.015	0.005	1.204
**176**	TB-only	1.944	0.831	0.063	0.004	0.012	0.004	0.001	0.801
**178**	TB-only	0.793	0.181	0.013	0.003	0.007	0.005	0.004	2.335
**180**	TB-only	4.331	1.960	0.106	0.015	0.089	0.020	0.003	1.781
**181**	TB-only	0	0	0	0.002	0.003	0.005	0.003	1.270
**182**	TB-only	0.047	0	0.004	0.005	0.009	0.010	0.004	1.535
**183**	TB-only	0.227	0.125	0.011	0.006	0.010	0.009	0.002	0.656
**188**	TB-only	0	0	0	0	0	0	0	0
**189**	TB-only	0.074	0	0.004	0.002	0.004	0.006	0.002	1.187
**190**	TB-only	1.978	0.862	0.038	0.009	0.024	0.010	0.003	1.293
**191**	TB-only	0	0	0.005	0.002	0.004	0.005	0.003	1.898
**192**	TB-only	1.644	0.463	0.025	0	0.005	0	0	0
**193**	TB-only	1.379	0.628	0.049	0	0.032	0	0	2.233
**194**	TB-only	5.749	1.674	0.048	0.009	0.047	0.020	0.003	1.646
**198**	TB-only	1.454	0.234	0.030	0.011	0.028	0.022	0.004	1.288
**46**	HIV-only	0.933	0.211	0.009	0.001	0.006	0	0	0.789

**48**	HIV-only	0.092	0.032	0.006	0.003	0.005	0.006	0.003	0.713
**57**	HIV-only	0.713	0.190	0.011	0.001	0.004	0	0	2.071
**62**	HIV-only	0.214	0.068	0.015	0.001	0.002	0	0	1.770
**63**	HIV-only	0.637	0.325	0.050	0.003	0.011	0.007	0.002	1.260
**77**	HIV-only	3.844	1.100	0.186	0.007	0.031	0.013	0.006	0.622
**80**	HIV-only	0.276	0.150	0.012	0	0.003	0	0	0
**13**	HIV/TB	0	0	0	0.007	0.012	0.015	0.004	1.053
**21**	HIV/TB	0.096	0.034	0.008	0.003	0.005	0.006	0.002	0.862
**92**	HIV/TB	0.991	0.346	0	0.015	0.031	0.009	0	0
**100**	HIV/TB	1.444	0.889	0.081	0.012	0.098	0.026	0.007	1.912
**103**	HIV/TB	2.882	0.076	0.007	0.015	0.023	0.028	0.012	1.078
**129**	HIV/TB	2.541	0.870	0.046	0.013	0.063	0.015	0.003	1.299
**145**	HIV/TB	0.602	0.124	0.017	0.005	0.010	0.013	0.005	1.199
**151**	HIV/TB	0	0	0.010	0.001	0.002	0	0	1.140
**186**	HIV/TB	0.815	0.374	0.020	0.003	0.011	0.005	0.003	0.978

Abbreviations: HC: healthy controls; HIV: human immunodeficiency virus; TB: tuberculosis; C2: acetyl-L-carnitine; C3: propionyl-L-carnitine; C5: isovaleryl-L-carnitine; C6: hexanoyl-L-carnitine; C8: octanoyl-L-carnitine; C10: decanoyl-L-carnitine; C12: dodecanoyl-L-carnitine; 5-HIAA: 5-hydroxyindoleacetic acid.

Table S7: Log-transformed mean abundances of amino acids and ratios subjected to statistical analysis

Compound	Mean log-transformed concentration (95% confidence interval)				Line graph*
	HC	HIV-only	TB-only	HIV/TB	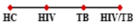
**Amino acids**					
α-Aminoadipic acid	-0.153 (0.373)	-1.109 (0.755) ↓	0.346 (0.228) ↑	-0.171 (0.703) ↓	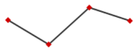
α-Aminobutyric acid	-0.041 (0.362)	-0.957 (0.723) ↓	0.217 (0.267) ↑	-0.099 (0.710) ↓	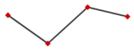
β-Alanine	-0.083 (0.362)	-0.582 (0.641) ↓	0.149 (0.294) ↑	0.071 (0.706) ↑	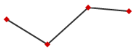
β-Aminoisobutyric acid	-0.109 (0.369)	-0.943 (0.563) ↓	0.185 (0.273) ↑	0.276 (0.692) ↑	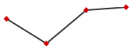
1-Methylhistidine	0.333 (0.263)	0.220 (0.921) ↓	-0.291 (0.317) ↓	-0.029 (0.747) ↓	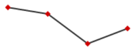
3-Methylhistidine	0.305 (0.328)	0.652 (0.502) ↑	-0.372 (0.292) ↓	0.102 (0.704) ↓	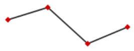
Alanine	-0.171 (0.434)	0.225 (1.120) ↑	0.122 (0.232) ↑	-0.121 (0.176) ↑	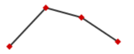
Anserine	0.038 (0.364)	-0.294 (0.695) ↓	0.004 (0.302) ↓	0.077 (0.684) ↑	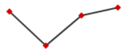
Arginine	-0.316 (0.392)	-0.831 (0.918) ↓	0.314 (0.238) ↑	0.337 (0.309) ↑	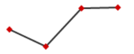
Argininosuccinic acid	-0.139 (0.378)	-0.976 (0.460) ↓	0.293 (0.271) ↑	-0.080 (0.588) ↑	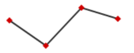
Asparagine	-0.209 (0.404)	-0.791 (1.086) ↓	0.338 (0.198) ↑	-0.181 (0.537) ↑	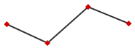
Carnosine	-0.132 (0.389)	-0.839 (0.603) ↓	0.358 (0.237) ↑	-0.506 (0.652) ↓	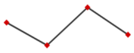
Cystathionine	-0.130 (0.360)	-0.774 (0.580) ↓	0.290 (0.283) ↑	-0.258 (0.644) ↓	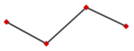

Cystine	0.089 (0.304)	-0.837 (1.040) ↓	0.123 (0.252) ↑	-0.224 (1.002) ↓	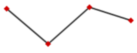
Ethanolamine	0.159 (0.205)	-1.043 (1.510) ↓	0.134 (0.173) ↓	-0.365 (1.245) ↓	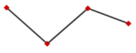
Glutamic acid	0.012 (0.361)	-1.113 (0.865) ↓	0.073 (0.279) ↑	0.490 (0.314) ↑	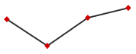
Glutamine	-0.060 (0.372)	-0.802 (0.775) ↓	0.202 (0.273) ↑	-0.082 (0.640) ↓	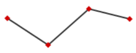
Glycine	0.498 (0.302)	0.558 (0.462) ↑	-0.297 (0.279) ↓	-0.851 (0.679) ↓	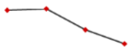
Histidine	0.291 (0.381)	0.144 (0.892) ↓	-0.174 (0.225) ↓	-0.354 (0.894) ↓	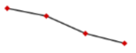
Homocitrulline	0.048 (0.375)	-0.721 (0.484) ↓	0.072 (0.293) ↑	0.062 (0.675) ↑	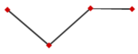
Hydroxykynurenine	-0.523 (0.302)	-1.014 (0) ↓	0.497 (0.255) ↑	0.385 (0.704) ↑	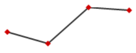
Hydroxylysine	-0.093 (0.357)	-0.087 (1.055) ↑	0.106 (0.291) ↑	-0.086 (0.565) ↑	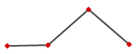
Isoleucine	-0.322 (0.371)	-1.272 (0.580) ↓	0.444 (0.209) ↑	0.111 (0.630) ↑	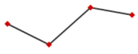
Kynurenine	-0.508 (0.346)	-1.033 (0.423) ↓	0.562 (0.202) ↑	0.051 (0.691) ↑	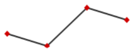
Leucine	-0.305 (0.391)	-0.923 (0.909) ↓	0.315 (0.217) ↑	0.368 (0.524) ↑	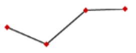
Lysine	0.009 (0.359)	-0.506 (1.167) ↓	0.116 (0.249) ↑	-0.166 (0.756)	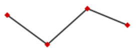
Ornithine	-0.234 (0.356)	-0.662 (0.723) ↓	0.275 (0.287) ↑	0.094 (0.580) ↑	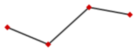
Phenylalanine	0.023 (0.319)	-0.500 (1.355) ↓	0.002 (0.275) ↓	0.300 (0.595) ↑	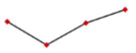
Phosphoethanolamine	-0.085 (0.380)	-1.014 (0.541) ↓	0.333 (0.243) ↑	-0.426 (0.683) ↓	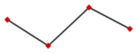
Pyroglutamic acid	-0.225 (0.377)	-0.142 (0.191) ↑	0.144 (0.333) ↑	0.256 (0.132) ↑	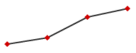
Saccharopine	-0.355 (0.344)	-1.092 (0) ↓	0.368 (0.272) ↑	0.435 (0.578) ↑	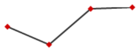
Serine	0.201 (0.325)	0.689 (0.224) ↑	-0.285 (0.325) ↓	0.050 (0.612) ↓	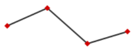
Taurine	-0.045 (0.348)	-0.100 (1.032) ↓	-0.006 (0.315) ↑	0.266 (0.328) ↑	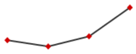
Threonine	0.063 (0.425)	-0.481 (1.145) ↓	0.092 (0.169) ↑	-0.268 (0.803) ↓	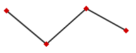
Tryptophan	0.103 (0.331)	-1.090 (1.262) ↓	0.245 (0.200) ↑	-0.638 (0.742) ↓	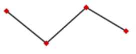
Tyrosine	0.198 (0.335)	-0.519 (1.342) ↓	-0.085 (0.285) ↓	0.084 (0.265) ↓	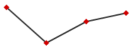
Valine	-0.003 (0.379)	-0.725 (0.869) ↓	0.094 (0.282) ↑	0.146 (0.486) ↑	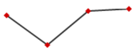
**Amino acid ratios**					
Glutamine/Glutamic acid	-0.101 (0.355)	-1.054 (0.521) ↓	0.198 (0.283) ↑	0.276 (0.645) ↑	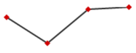
Kynurenine/Tryptophan	-0.510 (0.309)	-1.167 (0) ↓	0.597 (0.226) ↑	0.003 (0.694) ↑	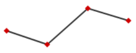
Phenylalanine/Tyrosine	-0.058 (0.355)	-0.587 (1.288) ↓	0.045 (0.268) ↑	0.457 (0.345) ↑	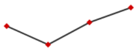
Saccharopine/Lysine	-0.518 (0.262)	-0.952 (0) ↓	0.466 (0.285) ↑	0.456 (0.731) ↑	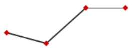

Abbreviations: HC: healthy controls; HIV: human immunodeficiency virus; TB: tuberculosis; Blue and ↓: decreased compared to healthy control group; Red and ↑: increased compared to healthy control group.

**These trendlines do not represent paired data relationships but rather serve as a general trend visualisation tool.*

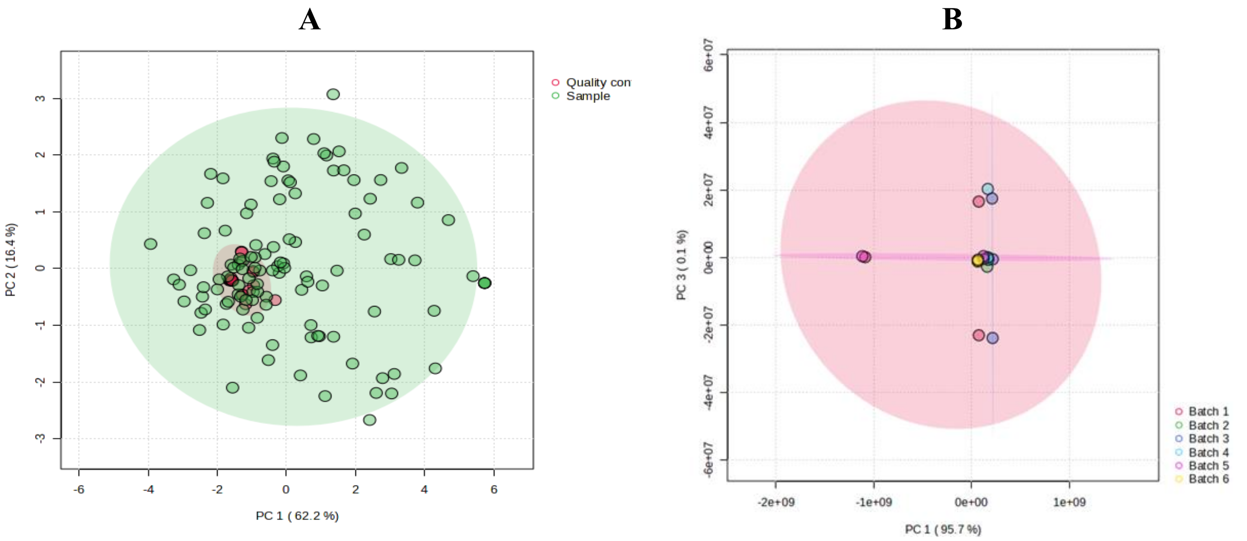

Figure S1: Principal component analysis (PCA) scores plot for acylcarnitine analysis. *PCA scores plots compare* ***(A)*** *quality control (QC) samples (in red) with patient urine samples (in green), and* ***(B)*** *individual QC samples for different batches (n=6), demonstrating data consistency and the absence of significant batch effects.*

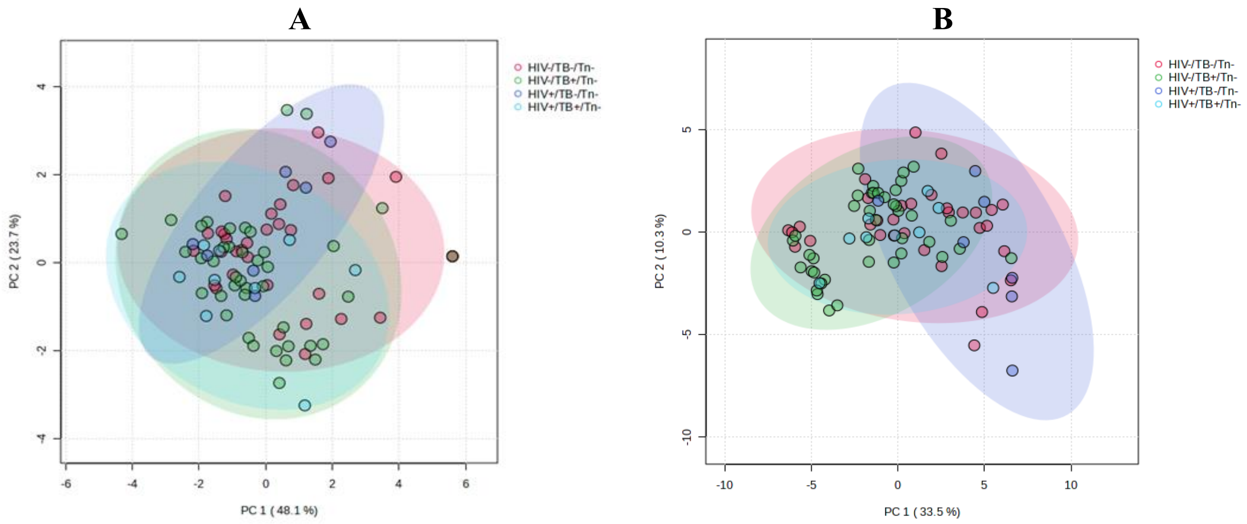

Figure S2: PCA scores plots for amino acid and acylcarnitine analyses. *PCA scores plots compare the healthy control group (HIV-/TB-/Tn-) with experimental groups based on* ***(A)*** *acylcarnitine analysis and* ***(B)*** *amino acid analysis, showing no significant separation, indicating similar metabolomic profiles across cohorts.*
